# Analysis of the Mechanical Properties of the AlSi7CrMnCu2.5 Alloy and Their Changes After Heat Treatment

**DOI:** 10.3390/ma18194586

**Published:** 2025-10-02

**Authors:** Pavel Kraus, Nataša Náprstková, Jaromír Cais, Sylvia Kuśmierczak, Klára Caisová, Anna Rudawska, Jan Sviantek

**Affiliations:** 1 Faculty of Mechanical Engineering, Jan Evangelista Purkyně University in Ústí nad Labem; Pasteurova 7, 400 96 Ústí nad Labem, Czech Republic; natasa.naprstkova@ujep.cz (N.N.); jaromir.cais@ujep.cz (J.C.); sylvia.kuszmierczak@ujep.cz (S.K.); klara.caisova@ujep.cz (K.C.); jan.sviantek@ujep.cz (J.S.); 2 Faculty of Mechanical Engineering, Lublin University of Technology, Nadbystrzycka 36, 20-618 Lublin, Poland; a.rudawska@pollub.pl

**Keywords:** alloy, heat treatment, hardening, chromium, mechanical properties

## Abstract

The article deals with the analysis of the mechanical properties of the newly designed aluminum alloy Al-Si7CrMnCu2.5. The research was carried out in order to map a new alloy with a certain addition of chromium and manganese from the point of view of mechanical properties and their changes after heat treatment (hardening, artificial aging) with defined parameters. Specifically, properties such as strength limit, yield strength, ductility, hardness, and microhardness were analyzed, both in the cast state and after heat treatment. The alloy was designed as an alternative to the standard Al-Si alloys already used in practice (AlSi7Mg, AlSi7Mg0.3, AlSi8Cu2Mn, AlSi8Cu3), which are mainly used in the production of engine parts and other components for the automotive and aviation industries. As can be seen from the presented results, the experimental AlSi7CrMnCu2.5 alloy exceeds the properties of the other selected alloys by tens of percent already in the cast state in many parameters. After heat treatment, the results achieved are comparable to the mentioned alloys, and in most cases, their values exceed them, especially in terms of ductility and hardness.

## 1. Introduction

Currently, the industrial world is in a phase of continuous technological development, which causes an increasing demand for suitable materials, which are central to high-quality and precise production. Aluminum alloys combine an excellent ratio of strength, weight, and high resistance to fatigue and corrosion [1], with an appropriately chosen ratio and type of alloying elements, which, as a result, enables the wide use of these materials in various areas of industrial activity [2].

The task of alloying elements in Al-Si alloys is to disrupt the negative effect of harmful elements, e.g., by binding them to other intermetallic phases [3], or to create “positive intermetallic phases” [4], which positively affect the properties of these alloys and create the possibility increase in strength properties (e.g., through heat treatment) [5], especially with regard to elements such as manganese and chromium [6].

The basis of the AlSi7CrMnCu2.5 alloy is the often-used foundry alloy AlSi7. Another alloying element is Cu in a content of approximately 2.5 wt.%. As is known, copper is an element that is necessary for precipitation hardening [7], and in this amount, it can be assumed [8] that during artificial aging, there should be a higher percentage, direct formation of GPII, and partially coherent precipitates [9], which should together ensure an increase in mechanical properties [10]. Chromium is present in the alloy in the range of 0.4 ÷ 0.5 wt.%. It has a positive effect on corrosion resistance [11] and, thanks to the formation of intermetallic phases, increases the strength properties of the alloy together with hardness [12]. This parameter can also be strengthened by the fact that Cr, up to 0.5% refines the grain [13]. Chromium binds iron to a certain extent from the alloy, similarly to manganese [14], which is present in the tested alloy with a content of 0.6 ÷ 0.7%. Like previous alloying elements, it increases mechanical properties such as strength and hardness and ensures their stability.

Heat treatment of aluminum alloys can be defined as a process in which the product or its part in a solid state is exposed to one or more heat cycles in order to achieve the desired structure [15]. Thanks to these structural changes, it is possible to achieve an increase in mechanical properties for hardenable alloys [16]. The quality control of castings after heat treatment is most often performed by measuring the hardness. Depending on the size of the casting and the size of the indentation or the required hardness unit, one of the standardized hardness measurement methods is chosen, while the measured value should correspond to the required or prescribed hardness value [17]. Hardening is, as is well known, a heat treatment consisting of a process of dissolution (homogenization), annealing, cooling at a supercritical rate, and subsequent aging. Cooling is usually carried out in water with a temperature of 20 to 70 °C. In order to prevent partial segregation of additives at the grain boundaries, it is necessary to include cooling immediately after holding at the solution annealing temperature [18]. A supersaturated solid solution is thermodynamically unstable and disintegrates [19]. In some alloys, the supersaturated solid solution breaks down already at ambient temperature; this process is referred to as natural aging [20]. In artificial aging, the process is accelerated by heating [21]. In general, the breakdown of a supersaturated solid solution is a diffusion process that begins with nucleation and the formation of coherent precipitates, the so-called Guinier–Preston zones (GP zones) [22]. The result is tension in the aluminum grid around the zones, which is an obstacle to the movement of dislocations, which is associated with a hardening effect and a subsequent change in mechanical properties. Each stage of dissolution of the solid solution has an effect on the physical and mechanical properties of the aluminum alloy. The greatest influence on strength is attributed to the metastable phase (GPI and GPII), which is coherent (cohesive) with the underlying solid solution [23].

The aim of this study is to investigate the mechanical properties of a newly designed AlSi7CrMnCu2.5 alloy in both as-cast and heat-treated conditions and to compare them with standardized Al-Si alloys widely used in industry. The focus is on tensile strength, ductility, hardness, and microstructural changes induced by heat treatment.

## 2. Experiment

The presented research is part of long-term activities in the field of aluminum alloy research and long-term cooperation of the Faculty of Mechanical Engineering JEPU in Ústí nad Labem with industry and production.

### 2.1. Experimental Material

The elements and their amount in the AlSi7CrMnCu2.5 alloy are selected so that the individual positive properties of the elements suppress or mitigate their other negative properties.

The properties of partially similar alloys Al-Si7Mg, AlSi7Mg0.3, AlSi8Cu2Mn, and AlSi8Cu3 are described within the ČSN EN 1706 standard [24]. Based on the knowledge of these properties, the chemical composition of these alloys can be determined, especially with regard to the copper content and its influence on the hardening phase of the heat treatment. According to this standard, it is also possible to determine the heat treatment conditions for the experiment, such as the temperature of recrystallization, annealing, and subsequent hardening, which were easily adapted to the alloying elements used and the possibilities of the workplace.

The melting of this alloy took place in an electric arc furnace; the melting temperature for the batch was 750 °C, the melt was cleaned with refining salt, and the subsequent casting temperature was 720 °C. The alloy was cast by manual gravity method into a metal mold with a complete sprue system pre-heated to 350 °C.

During melting, samples were taken from the melt in the foundry for spectrometric analysis, in which the exact chemical composition of the alloy was checked on an optical emission spectrometer (Bruker Q4 Tasman, Kalkar, Germany).

The specific complete chemical composition of the cast AlSi7CrMnCu2.5 alloy is shown in Table 1.

### 2.2. Heat Treatment

**Figure 1:**
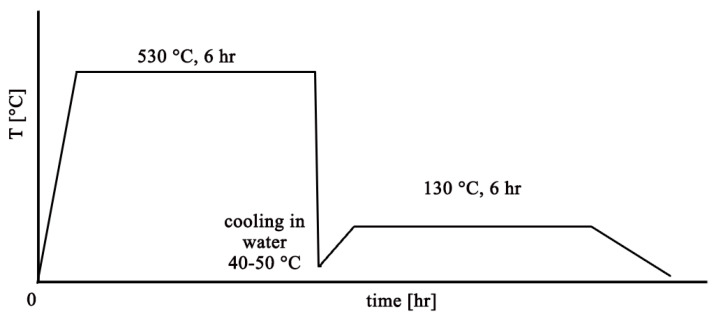
Scheme of heat treatment of the AlSi7CrMnCu2.5 alloy.

The temperature regimes used for purposes of this experiment are based on the EN 1706:2020 standard [24], in which suitable heat treatment temperatures are given for the above-mentioned alloys, with which AlSi7CrMnCu2.5 is compared. Based on the available literature sources, the most frequently used artificial aging temperature for the aforementioned similar alloys is 130 ÷ 150 °C [25,26]. The diagram of the complete regime of the selected heat treatment is shown in Figure 1.

Solution annealing was performed in an induction furnace LAC K 70/13 (manufactured by the LAC company, Židlochovice, Czech Republic). The start-up time required to reach the solution annealing temperature was approx. 1 h. The duration at the dissolution temperature of 530 °C lasted 6 h, and then the samples were cooled in water at a temperature of 40 ÷ 50 °C. The time of moving the samples from the oven to the water for cooling was always approx. 20 s. Immediately (within seconds) after cooling, the samples moved to the second cycle of heat treatment—artificial aging. Precipitation took place in a Binder dryer (manufactured by the DONAU LAB company, Prague, Czech Republic), which was always pre-heated to a curing temperature of 130 °C according to the applied artificial aging regime. The heating time to the curing temperature was always approximately 1 h due to the lower performance of the device. The temperature duration for each of the applied artificial aging regimes was 6 h. Subsequently, the samples cooled on their own in the dryer to ambient temperature for approximately 2 h.

For the purposes of evaluating the experiment, the heat-treated alloy AlSi7CrMnCu2.5 is marked as HT (heat-treated) and in cast condition CC (cast condition).

## 3. Results

In order to investigate the mechanical properties of the AlSi7CrMnCu2.5 alloy, a static tensile test was performed to analyze the strength limit of the material R_m_, its ductility A, the maximum force acting on the sample *F_max_*, and the contractual yield strength *R_p0.2_*. Furthermore, its complex hardness at lower load HV1, microhardness of solid solution HV0.1, and hardness according to Brinell HB were measured.

### 3.1. The Tensile Test

**Figure 2:**
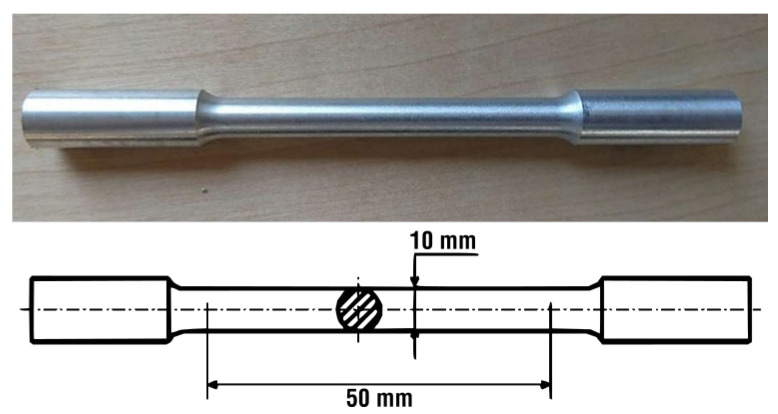
Tensile specimen.

The static tensile test is one of the basic tests for determining the mechanical properties of metals. To determine the mechanical properties of the AlSi7CrMnCu2.5 alloy, a HEGEWALD & PESCHKE INSPECT 250 universal blasting machine (manufactured by the HEGEWALD & PESHKE company, Nossen, Germany) with a strain gauge was used for the experiment. The samples (see Figure 2) for the tensile test were machined into the shape of standardized test specimen rods with a circular cross-section of 10 mm in diameter and a test section length of 50 mm, determined according to ČSN EN ISO 6892-1 [27].

The test specimens were loaded by uniaxial static stress and tension at an ambient temperature of 21 °C until complete rupture. The loading rate of the tested specimens was 10 mm/min, without a prior initial preload. During the static tensile test, the stress dependence curve on the proportional elongation was recorded. The results of the static tensile test of samples without heat treatment, i.e., in the cast state (CC), are shown in Table 2.

**Figure 3:**
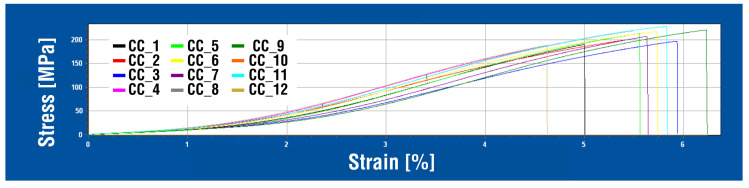
Stress–strain curves—CC samples.

The static tensile test curves of individual samples of the AlSi7CrMnCU2.5 alloy in the cast state (CC) are shown in the multiple diagram in Figure 3. The curves of the AlSi7CrMnCu2.5 alloy were created in the HEGEWALD & PESCHKE tensile testing machine software LabMaster v.3.0.9.17 (manufactured by Hegewald & Peschke Meß- und Prüftechnik GmbH, Nossen, Germany) and do not show significant yield strength or other abnormalities; their general shape is almost identical for each test bar from the CC series.

The results of the static tensile test of the samples after heat treatment (HT) are shown in Table 3.

**Figure 4:**
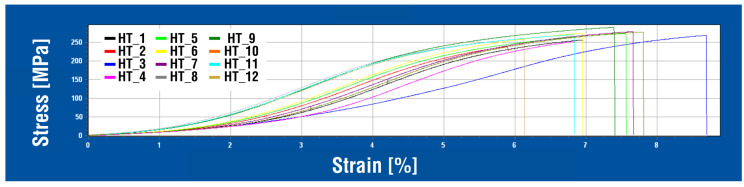
Stress–strain curves—HT samples.

The static tensile test curves of individual samples of the AlSi7CrMnCU2.5 alloy after heat treatment (HT) are shown in the multiple diagram in Figure 4. The curves of the AlSi7CrMnCu2.5 alloy were created in the HEGEWALD & PESHKE tensile testing machine software and do not show significant yield strength or other abnormalities; their general shape is almost identical for each test bar from the HT series. The curves have a gradually increasing character without a significant boundary of the transition between elastic and plastic deformation.

### 3.2. Evaluation of Hardness and Microhardness

**Figure 5:**
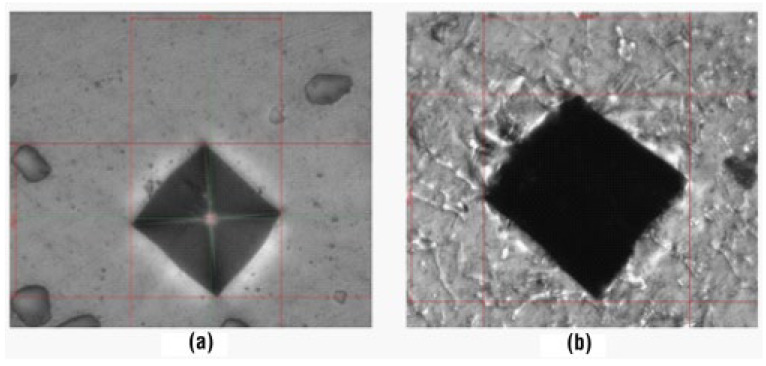
Microhardness and hardness measurements: (**a**) HV0,1—solid solution (diagonal 42 µm), (**b**) HV1—complex surface (diagonal 142 µm).

Another part of the experimental analysis was to perform hardness and microhardness measurements. Samples were taken from the cast bars prior to machining to the final stage of tensile test specimens and metallographically prepared for analysis. On each of the samples, the microhardness of the solid solution HV0.1 and the complex hardness at low load HV1 as a whole (Figure 5) were measured using a FUTURE-TECH FM-300 microhardness tester (manufactured by the FU-TURE-TECH CORP., Kawasaki, Japan). Taking into account the circumstance that the tested AlSi7CrMnCu2.5 alloy will be further compared with the standardized alloys AlSi7Mg, AlSi7Mg0.3, AlSi8Cu2Mn, and AlSu8Cu3; the value of which is given in the standard according to the Brinell hardness scale. The Brinell hardness was also measured for the HT and CC samples on the ERNST AT 250 X device (manufactured by the CISAM-ERNST company, Induno Olona, Italy) by pressing a tungsten ball with a diameter of 2.5 mm into the material with a force of 62.5 kgf for 10 s.

The results of Vickers HV1 low load hardness analysis of the CC and HT samples are shown in Table 4.

For the HT and CC samples, the microhardness of the HV0.1 solid solution was also measured to determine the hardness before and after the diffusion of copper and other alloying elements into the Al-Si7CrMnCu2.5 alloy matrix. The results of measuring the microhardness HV0.1 of the solid solution are presented in Table 5.

The results of the hardness measurement according to Brinell HB are shown in Table 6 and are correlated with the results of the hardness measurement according to Vickers HV1.

### 3.3. Microstructure Analysis

The reason for the increase in mechanical properties can be explained by the change in the microstructure after heat treatment. For all microstructure analyses, an OLYMPUS LEXT OLS 500 (manufactured by the OLYMPUS CORP., Tokyo, Japan) confocal laser microscope and a TESCAN VEGA 3 (manufactured by the TESCAN GROUP, Brno, Czech Republic) electron microscope were used.

**Figure 6:**
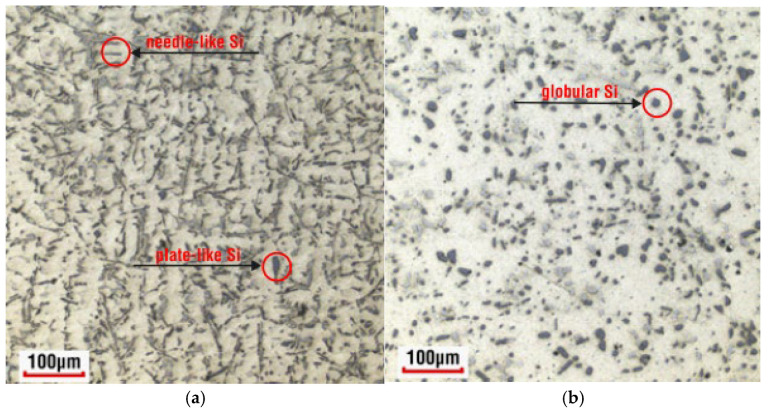
Microstructure of the CC (**a**) and HT (**b**) samples.

Figure 6 presents a comparison of the microstructure of the AlSi7CrMnCu2.5 alloy in the as-cast state (Figure 6a) and after heat treatment (Figure 6b). As can be seen from the image, the needles and plates of eutectic silicon (dark gray formations in image (Figure 6a)) were transformed into smaller globular formations (Figure 6b) due to heat treatment. Also, other (Cu-Si, Cr-Mn, or polycomponent) intermetallic phases changed their shape by the heat treatment process.

**Figure 7:**
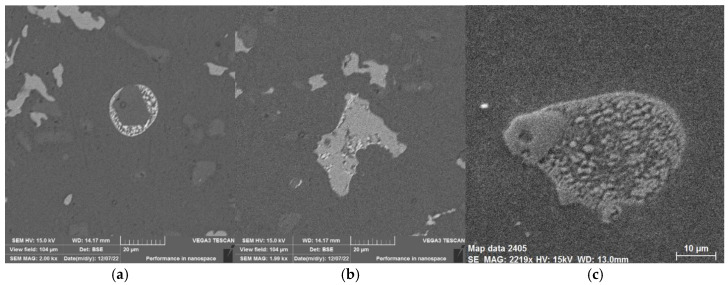
Phases by SEM analysis: (**a**) Cu-Si phase, (**b**) Cr-Mn phase, (**c**) polycomponent phase [4].

Another reason for the change in mechanical properties after heat treatment was the precipitation of elements into a solid solution and the formation of a number of phases, which are shown in the SEM analysis images [4] in Figure 7.

**Figure 8:**
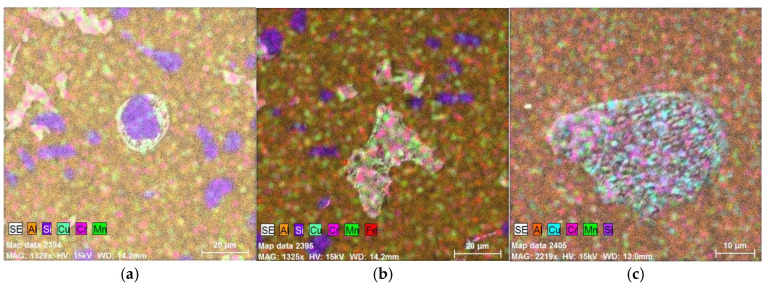
EDX analysis: (**a**) Cu-Si phase, (**b**) Cr-Mn phase, (**c**) polycomponent phase.

Elemental mapping (see Figure 8) of identified intermetallic phases (see Figure 7) has been conducted via EDX analysis using a TESCAN VEGA 3 electron microscope (manufactured by TESCAN GROUP, a.s., Brno, Czech Republic) with an EDX BRUKER analyser (manufactured by BRUKER Corp., Billerica, MA, USA).

### 3.4. Comparison of Mechanical Properties

When compared with the alloys widely used in the automotive industry, such as AlSi7Mg, AlSi7Mg0.3, Al-Si8Cu2Mn, and AlSi7Cu2, it can be seen that the experimental alloy AlSi7CrMnCu2.5 usually has tens of percent higher all analyzed mechanical properties in the cast state (CC) [16], as can be seen from Table 7.

The selected properties of the AlSi7CrMnCu2.5 alloy after heat treatment (HT) are comparable to the alloys listed in Table 6, and in most cases, their values exceed them, especially in terms of ductility, hardness, and contractual yield strength.

## 4. Discussion

The mechanical testing demonstrates that AlSi7CrMnCu2.5 outperforms conventional Al-Si alloys in both the as-cast and heat-treated states. The tensile test results indicate that the new alloy has approximately 20–30% higher strength and ductility after heat treatment compared to its cast state, which can be attributed to microstructural refinement and precipitation processes.

The improvement in ductility is closely linked to the transformation of eutectic silicon morphology. Heat treatment caused the originally needle-like and plate-like silicon structures to globularize, reducing stress concentrations and enabling higher plastic deformation before fracture. In parallel, precipitation of Cu- and Mn/Cr-containing phases increased matrix strengthening, explaining the higher yield strength and hardness.

The hardness and microhardness results confirm these findings, showing consistent increases across all scales. The rise in HV0.1 suggests effective diffusion of alloying elements into the Al matrix, while the increases in HV1 and HB demonstrate the overall improvement of bulk mechanical resistance.

In addition to the observed globularization of eutectic silicon and the formation of secondary phases, other mechanisms likely contribute to the improved mechanical properties. These include solid solution strengthening during solution treatment, increased dislocation density after quenching, and the homogenization of the microstructure. Together, these mechanisms explain the simultaneous increase in strength, hardness, and ductility after heat treatment.

When compared with standardized alloys, AlSi7CrMnCu2.5 shows clear advantages in the as-cast state, particularly in yield strength and ductility, which are often weaknesses in conventional alloys. After heat treatment, the alloy remains competitive, achieving strength values close to or exceeding those of standard alloys while offering superior ductility, which can be useful within the automotive industry (such as cylinder heads, blocks).

## 5. Conclusions

This article focused on the research of selected properties of the newly developed aluminum alloy AlSi7CrMnCu2.5 as an alternative to the already used in practice aluminum alloys intended for hardening, with the acceptance of available world research that takes into account a similar field of material sciences.

Samples of the newly developed alloy were subjected to heat treatment followed by artificial aging (HT), with a series of samples left in the as-cast condition (CC) for comparison purposes.

Based on the experiments carried out and the analyzed quantities, the following can be stated:

- The newly developed AlSi7CrMnCu2.5 alloy shows significantly higher strength, yield strength, ductility, and hardness than selected standard Al-Si alloys in the as-cast state.
- Heat treatment (solution annealing and artificial aging) further improves mechanical properties, increasing tensile strength by ~25% and ductility by ~30%.
- Hardness values (HV1, HV0.1, HB) consistently increase after heat treatment, reflecting effective precipitation strengthening.
- Microstructural analysis confirms that globularization of eutectic silicon and precipitation of secondary phases are key factors in property improvements.

## Figures and Tables

**Table 1:** The chemical composition of alloy AlSi7CrMnCu2,5 (Q4 TASMAN).

Chemical Composition [wt.%]								
Si	Fe	Cu	Mn	Cr	Mg	Ti	Sr	Al
6.892	0.263	2.487	0.612	0.491	0.041	0.052	0.001	base

**Table 2:** Results of the tensile test—CC samples.

Results of the Tensile Test—CC Samples				
Sample	*R_m_* [MPa]	*R_p_*_0.2_ [MPa]	*F_max_* [kN]	*A* [%]
CC_1	189.6	189.6	14.8	5.0
CC_2	199.4	179.3	15.6	5.4
CC_3	196.6	181.1	15.4	5.9
CC_4	192.8	190.1	15.1	4.7
CC_5	212.7	200.8	16.7	5.5
CC_6	217.0	192.5	17.0	5.7
CC_7	206.9	201.8	16.2	5.6
CC_8	218.2	196.8	17.1	5.5
CC_9	220.4	203.0	17.3	6.2
CC_10	187.9	187.6	14.7	4.6
CC_11	227.7	204.1	17.8	5.8
CC_12	197.6	180.4	15.4	5.1
**Ø**	**205.6**	**192.3**	**16.1**	**5.4**

**Table 3:** Results of the tensile test—HT samples.

Results of the Tensile Test—HT Samples				
Sample	*R_m_* [MPa]	*R_p_*_0,2_ [MPa]	*F_max_* [kN]	*A* [%]
HT_1	255.7	225.5	20.0	6.9
HT_2	248.6	221.9	19.1	6.1
HT_3	268.4	257.5	21.0	8.7
HT_4	252.2	228.9	19.8	6.8
HT_5	273.5	223.1	21.4	7.5
HT _6	274.3	231.1	21.5	6.9
HT _7	278.7	230.9	21.8	7.6
HT _8	259.3	220.0	20.3	6.1
HT _9	290.0	227.1	22.7	7.4
HT _10	259.5	224.9	20.3	6.1
HT _11	270.1	220.7	21.2	6.8
HT _12	277.6	232.3	21.8	7.8
**Ø**	**267.4**	**228.7**	**21.0**	**7.1**

**Table 4:** Results of the hardness at low load HV1.

HV1		
Measure	HT	CC
1.	109	91
2.	114	88
3.	115	93
4.	112	91
5.	115	88
6.	117	88
7.	118	90
8.	116	92
9.	113	91
10.	109	90
**Ø**	**114**	**90**

**Table 5:** Results of the microhardness HV0.1.

HV0.1 Solid Solution		
Measure	HT	CC
1.	109	84
2.	108	83
3.	109	85
4.	110	82
5.	109	84
6.	109	84
7.	109	84
8.	108	84
9.	109	83
10.	111	83
**Ø**	**109**	**84**

**Table 6:** Hardness measurement according to Brinell HB.

HB		
Measure	HT	CC
1.	115	84
2.	110	88
3.	109	83
4.	112	85
5.	109	85
6.	110	84
7.	116	82
8.	117	86
9.	111	87
10.	112	84
**Ø**	**112**	**85**

**Table 7:** Comparison of several alloys‘ mechanical properties [16].

Property/Alloy	AlSi7CrMnCu2.5	AlSi7Mg	AlSi7Mg0.3	AlSi8Cu2Mn	AlSi8Cu3
**Cast condition**					
***R_m_*** **[MPa]**	206	170	170	180	170
***R_p_*** **_0.2_** **[MPa]**	192	90	90	120	100
***A*** **[%]**	5.4	2.5	2.5	1	1
**HB (HV) [-]**	85 (90)	50 (54)	50 (54)	70 (74)	75 (80)
**Heat-treated condition**					
***R_m_*** **[MPa]**	267	260	290	265	280
***R_p_*** **_0.2_** **[MPa]**	229	220	210	210	250
***A*** **[%]**	7.1	1	1	1	1
**HB (HV) [-]**	112 (114)	90 (95)	90 (95)	95 (100)	95 (100)

## Data Availability

The original contributions presented in this study are included in the article. Further inquiries can be directed to the corresponding authors.
